# Assessing the disturbance potential of small unoccupied aircraft systems (UAS) on gray seals (*Halichoerus grypus*) at breeding colonies in Nova Scotia, Canada

**DOI:** 10.7717/peerj.4467

**Published:** 2018-03-19

**Authors:** Lauren Arona, Julian Dale, Susan G. Heaslip, Michael O. Hammill, David W. Johnston

**Affiliations:** 1 Division of Marine Science and Conservation, Nicholas School of the Environment, Duke University Marine Laboratory, Beaufort, NC, United States of America; 2 Institut Maurice-Lamontagne/Maurice Lamontagne Institute, Pêches et Océans Canada/Fisheries and Oceans Canada, Mont-Joli, QC, Canada

**Keywords:** Disturbance, Marine mammals, Grey seal, UAS, Drone, Unoccupied aircraft system, Acoustic, Noise, Abundance estimation, Aerial surveys

## Abstract

The use of small unoccupied aircraft systems (UAS) for ecological studies and wildlife population assessments is increasing. These methods can provide significant benefits in terms of costs and reductions in human risk, but little is known if UAS-based approaches cause disturbance of animals during operations. To address this knowledge gap, we conducted a series of UAS flights at gray seal breeding colonies on Hay and Saddle Islands in Nova Scotia, Canada. Using a small fixed-wing UAS, we assessed both immediate and short-term effects of surveys using sequential image analysis and between-flight seal counts in ten, 50 m^2^ random quadrats at each colony. Counts of adult gray seals and young-of-the-year animals between first and second flights revealed no changes in abundance in quadrats (matched pair *t*-test *p* > 0.69) and slopes approaching 1 for linear regression comparisons (*r*^2^ > 0.80). Sequential image analysis revealed no changes in orientation or posture of imaged animals. We also assessed the acoustic properties of the small UAS in relation to low ambient noise conditions using sound equivalent level (Leq) measurements with a calibrated U-MIK 1 and a 1/3 octave band soundscape approach. The results of Leq measurements indicate that small fixed-wing UAS are quiet, with most energy above 160 Hz, and that levels across 1/3 octave bands do not greatly exceed ambient acoustic measurements in a quiet field during operations at standard survey altitudes. As such, this platform is unlikely to acoustically disturb gray seals at breeding colonies during population surveys. The results of the present study indicate that the effects of small fixed-wing UAS on gray seals at breeding colonies are negligible, and that fixed-wing UAS-based approaches should be considered amongst best practices for assessing gray seal colonies.

## Introduction

The use of small unoccupied aircraft systems (UAS) for terrestrial commercial and research applications is now wide-spread in ecological science and wildlife management (Anderson & Gaston, 2013; Linchant et al., 2015). These devices are used to assess agricultural performance through a combination of visible and multispectral imagery (Zhang & Kovacs, 2012). Small UAS are used to assess environmental compliance in mining operations and to study the terrestrial habitats and abundance of wildlife (Koh & Wich, 2012; Linchant et al., 2015). These devices are also being used to study marine systems, including coastal habitat surveys (Mancini et al., 2013; Seymour et al., 2017b), oceanographic studies (Inoue & Curry, 2004; Elarab et al., 2015), and surveys of marine megavertebrates including marine mammals, sea turtles, and seabirds (Koski et al., 2009; Hodgson, Kelly & Peel, 2013; Bevan, Wibbels & Najera, 2015; Durban et al., 2015; Sykora-Bodie et al., 2017; Seymour et  al.,  2017a).

Dedicated surveys of animals are required to develop estimates of species abundance and distribution, and these data are fundamental for ecological studies and applied research for management and conservation purposes (Krebs, Hickman & Hickman, 1994; Lancia et al., 2005). Traditional surveys of many animals are conducted using human occupied helicopters or fixed-wing aircraft. These approaches can pose significant human risk (Sasse, 2003) and can be costly for some species and study areas (Vermeulen et al., 2013), In some cases considerable disturbance can occur when collecting aerial imagery with occupied aircraft (reviewed in Richardson et al., 1995). Satellite and other earth observation imagery provide new opportunities to assess wildlife populations without disturbance (Moxley et al., 2017; McMahon et al., 2014); however, these methods often cannot resolve smaller animals (Fretwell & Trathan, 2009), and are hampered by atmospheric interference from clouds. The use of UAS can, in some situations, overcome these constraints while presenting opportunities to reduce costs and risk (Linchant et al., 2015).

However, the operational use of UAS for imaging individual animals and surveying animal colonies requires careful assessment to determine efficacy and accuracy in relation to traditional methods, as well as determining their potential for disrupting the behavior of target species (McEvoy, Hall & McDonald, 2016). Recent studies have addressed potential cryptic disturbance of wildlife from UAS (e.g., heart rate changes, see Ditmer et al., 2015), and some studies have focused on the effects of small multirotor UAS on marine mammals (Pomeroy, O’Connor & Davies, 2015; Smith et al., 2016). However, the most recent review of disturbance effects of UAS on marine mammals revealed that little is known about the responses of most species to either fixed-wing or multicopter UAS (Smith et al., 2016).

The present study assesses the potential for small electric fixed-wing UAS to disturb phocid seals at breeding colonies in Nova Scotia, Canada. We used acoustic data and animal counts from aerial imagery to assess whether (1) a small electric fixed-wing UAS is likely to be detected by gray seal adults and young-of-the-year (YOY) animals and (2) whether gray seal adults and YOYs at breeding locations in Canada are startled or stampeded by UAS fly-overs.

**Figure 1:**
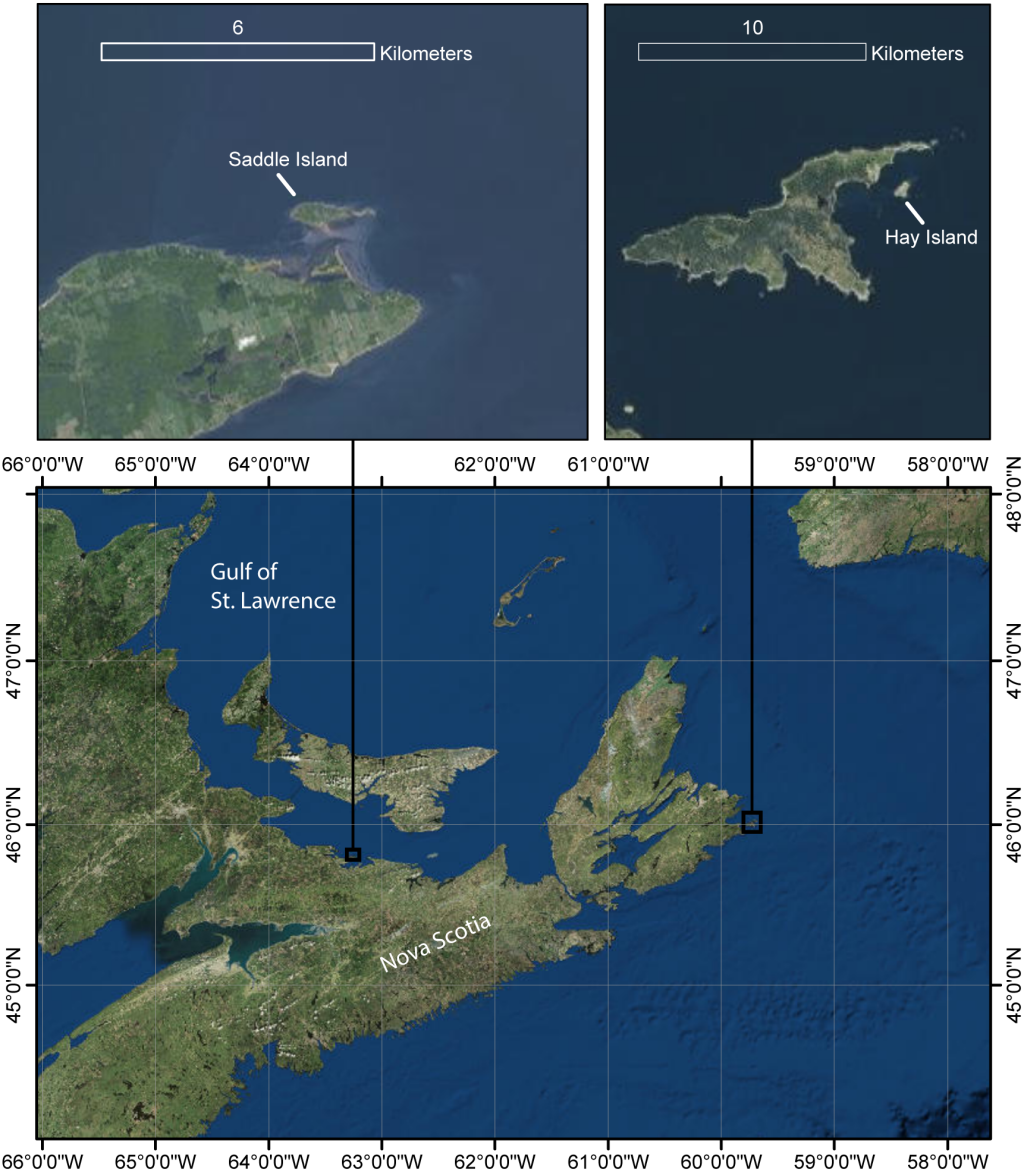
Map of study area. Locations of grey seal (*Halichoerus grypus*) breeding colonies on Saddle and Hay Island, Nova Scotia, Canada surveyed with unoccupied aircraft systems (UAS) during January 29–February 2, 2015.

## Methods

### Study location

Surveys of seals were conducted during January 29 to February 2, 2015 at Hay Island and Saddle Island, two gray seal breeding locations in Nova Scotia, Canada (Fig. 1) as part of a larger projecting assessing the utility of UAS to assess abundance of seals (e.g., Hammill et al., 2017; Seymour et al., 2017b; Seymour et al., 2017a). Acoustic measurements were collected at an isolated field in Beaufort, NC, USA.

### Small unoccupied aircraft system

We used the eBee, a modular fixed-wing UAS produced by the company senseFly. The eBee has a light-weight foam airframe powered by a single rear-mounted brushless electric motor powered by a lithium polymer battery. They have a wing-span of 96 cm and weigh 0.7 kg.

During surveys, the eBee UAS followed a pre-programmed three-dimensional flight path guided by a precision GPS sensor, a high-resolution barometer, ground-sensing camera and wind-speed indicators. Failsafe logic within the autopilot was set to return the UAS to the landing zone if it experienced anomalies in sensor performance or extreme wind conditions, and it telemetered flight data to the operator over UHF frequencies in real-time. The instrument was launched by hand and recovered after a linear approach/landing at a predetermined 10 m radius region.

### Acoustic data

Acoustic recordings of environmental sounds were made with a calibrated microphone and sound-level meter for both control periods (no UAS) and periods when the UAS was circling over the microphone at typical survey altitudes of 75 m and 85 m. Specifically, we collected 60 s unweighted equivalent sound pressure level (Leq) measurements (expressed in dB re 20 uPa 21 m) at standard 1/3rd octave bands from 20 Hz to 20 kHz using a calibrated UMIK-1 connected to an Apple iPad running Faber Acoustical SoundMeter Pro (Kardous & Shaw, 2014). We also collected Leq measurements (as above) of the sound of the eBee at full throttle and ready for takeoff from four orientations: 1 m in front of the aircraft, 1 m on either side of the aircraft, and 1 m behind the aircraft. Leq measurements capture averaged sound levels at a location, and present a standardized way to assess how relatively constant sounds, such as aircraft or industrial noise, and most ambient noise, change during experimental procedures or natural experiments (Pater, Grubb & Delany, 2009).

A full recording of the eBee starting up was also made with the UMIK-1 connected to the Apple iPad using the Tascam sound recorder application. The recording was imported into Raven Pro sound processing software for visualization. All sound measurements were made on a calm day (approx. 2.5–3.5 m per second wind) in an isolated field.

The Department of Fisheries and Oceans, Canada provided full approval for this purely observational research. All observational flights were conducted according to Canadian small UAS rules (Exemption from Sections 602.41 and 603.66 of the Canadian Aviation Regulations, see http://www.tc.gc.ca/civilaviation/regserv/affairs/exemptions/docs/en/2880.htm). All UAS flights for acoustic measurements were made under an FAA section 333 Exemption 12656 and associated blanket Certificate of Authorization (COA) awarded to Duke University.

### Seal count data and sequential imagery assessment

The senseFly eBee was flown at Saddle and Hay Island, Nova Scotia as part of an experiment to assess the utility of UAS for gray seal population assessment purposes (Hammill et al., 2017; Seymour et al., 2017b; Seymour et al., 2017a). Both colonies were undisturbed by human activity preceding drone surveys. Two flights at each colony were conducted at typical population assessment survey altitudes between 75–80 m to assess disturbance (variation in altitude during flight is due to wind gusts and undulating flight path of the eBee drone). Each flight lasted between 20–25 min (to cover the entire colony) and RGB imagery was captured at 3.3 cm ground sampling resolution. Images were collected sequentially with 85% longitudinal overlap, resulting in a 3.5 s gap between each photo taken. All flights were conducted according to Canadian small UAS rules (Exemption from Sections 602.41 and 603.66 of the *Canadian Aviation Regulations*) and under permit by Fisheries and Oceans, Canada.

The data collected by the eBee UAS was processed using Postflight Terra Version 4.0.1 software (senseFly, Switzerland) to create orthomosaics of both colonies. Each orthomosaic was corrected for any inconsistencies (e.g., movements of animals) and then then imported into the iTag software package to count the number of adult seals and young-of-the-year (YOY) seals present on each flight for population assessment studies (see Hammill et al., 2017; Seymour et al., 2017a; Johnston et al., 2017).

For the present disturbance assessment, ten 50 m^2^ quadrats were chosen randomly from a grid of each colony and the number of seals in each quadrat enumerated from the initial flight. These same quadrats were then enumerated for the second flight over the colony, to look for changes in the density or distribution of animals that may have occurred between flights. Linear regressions of seal counts within each 50 m^2^ quadrat between the first and second surveys were done in JMP 11 Pro. Paired *t*-tests of seals counts in each quadrat from the first and second surveys were also conducted to assess for any statistical changes in numbers between flights that would indicate that animals were flushed from specific locations by the overflights.

Images from the first survey line at each undisturbed survey location were also assessed for startle effects by examining the locations and orientation of individual seals observed in sequential images as the UAS flew over. While detailed ethograms of gray seals at these colonies during the breeding season do not exist, the large longitudinal overlap of imagery taken by the UAS (75–85%), allowed us to repeatedly image individual adult and YOY seals (images taken 3.5 s apart) to assess whether individual animals were immediately startled by the aircraft overhead. Startle effects were defined as directed movements of more than 2 body lengths in any direction, or obvious changes in body posture including body arching, rolling over, or changes in the direction the seals were facing. Assessment of startle effects was conducted by importing pairs of sequential images into Photoshop and examining the relative locations, orientation and posture of all seals available in image pairs.

## Results

**Figure 2:**
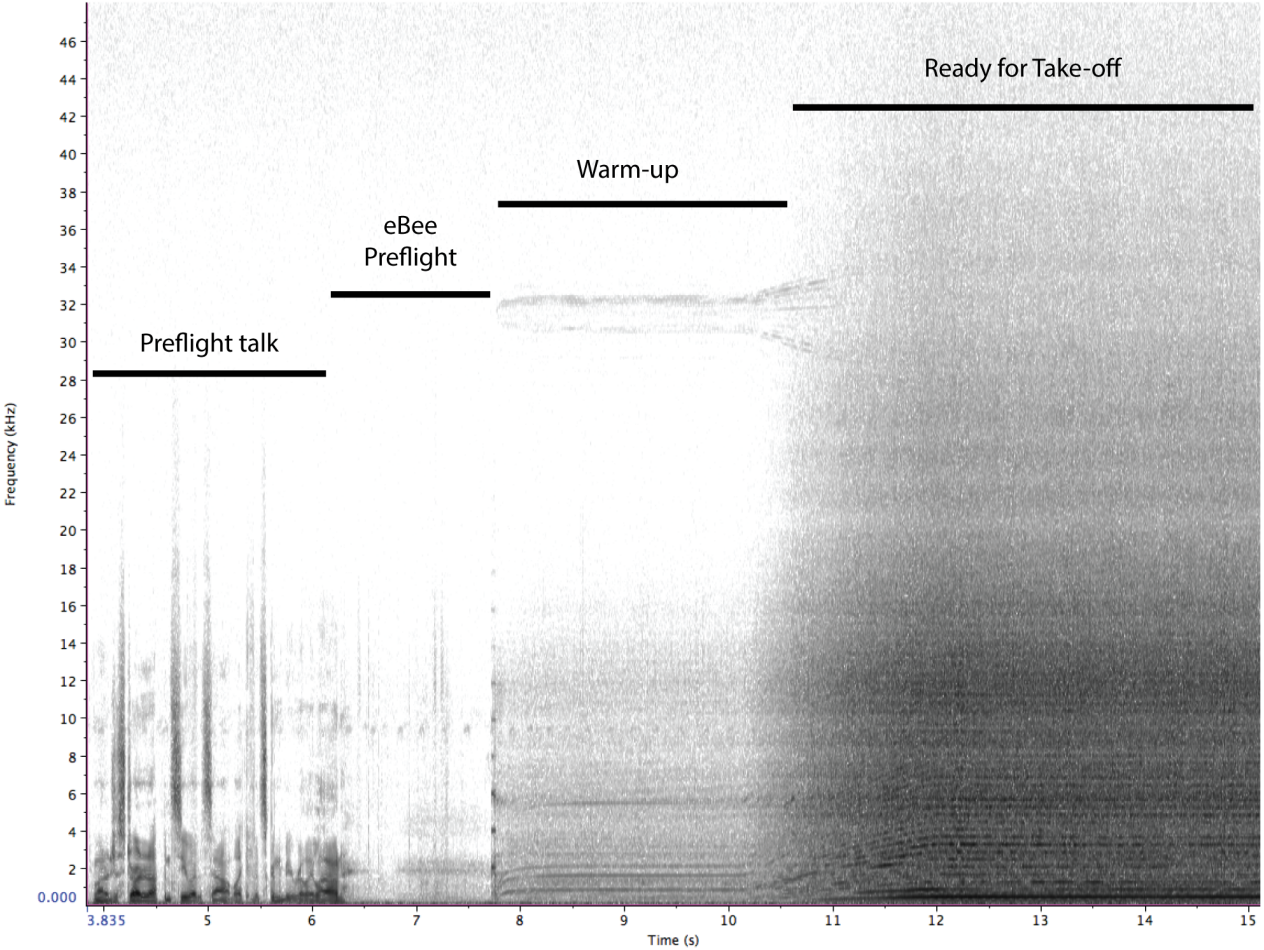
A spectrogram of the sounds produced by the eBee UAS during launch. This includes pre-flight talk by human operators and the launch sounds of the UAS itself.

**Figure 3:**
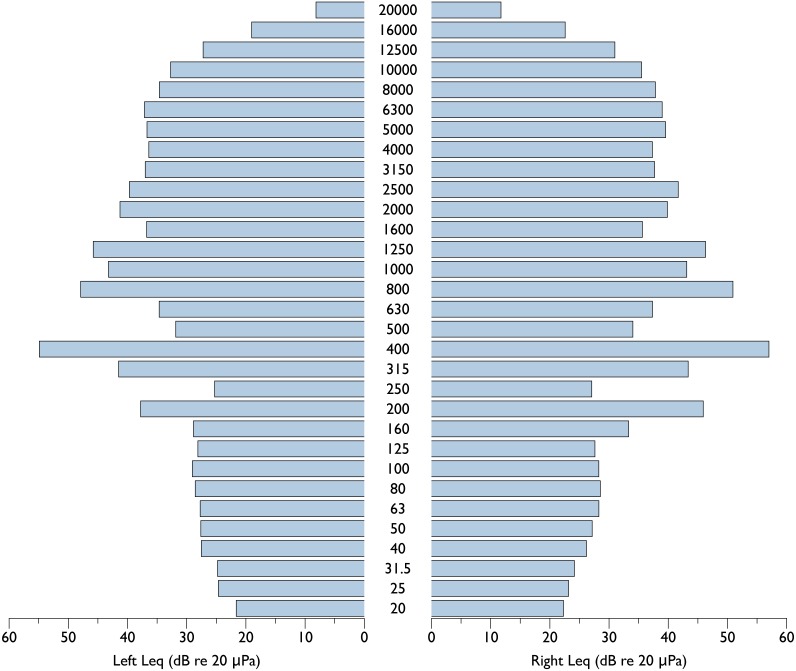
Equivalent sound pressure levels at 1/3 octave bands for the eBee small UAS on both left and right sides. Equivalent sound pressure levels at 1/3 octave bands (dB re 20 µPa @ 1 m) for the eBee small unoccupied aircraft system (UAS) at full throttle measured at 1 m on both left and right sides.

**Figure 4:**
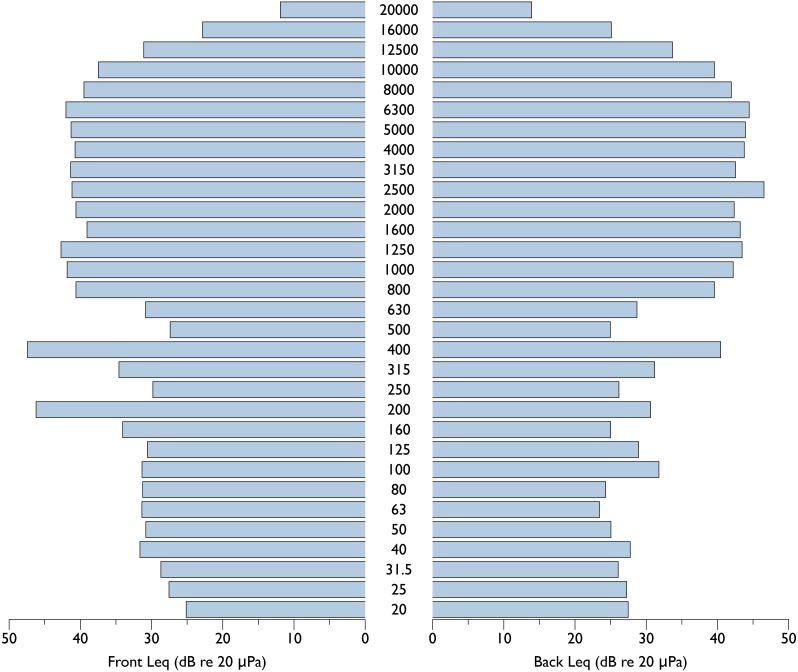
Equivalent sound pressure levels at 1/3 octave bands for the eBee small UAS in front and behind of the aircraft. Equivalent sound pressure levels at 1/3 Octave bands (dB re 20 µPa @ 1 m) for the eBee small unoccupied aircraft system (UAS) at full throttle measured at 1 m in front and behind of the aircraft.

A spectrogram of sounds produced by the eBee UAS is provided in Fig. 2. This figure depicts sounds associated with the take-off sequence, including preflight talk by operators, preflight tests of control surfaces and spin up of engine for launch. The engine is loudest at launch, and the aircraft reduces RPMs after gaining survey altitude. Unweighted equivalent sound pressure level (Leq) measurements (expressed in dB re 20 uPa @ 1 m) at standard 1/3rd octave bands for the eBee at full throttle and obtained from 4 orientations (front, back, left and right) are presented in Figs. 3 and 4. Both left/right and front/back recordings present symmetrical patterns, with slightly louder Leq values in front of the aircraft compared to behind. At takeoff, the eBee produces its loudest sounds above 160 Hz, with values approaching 60 dB re 1 uPa at 200 and 400 Hz.

**Figure 5:**
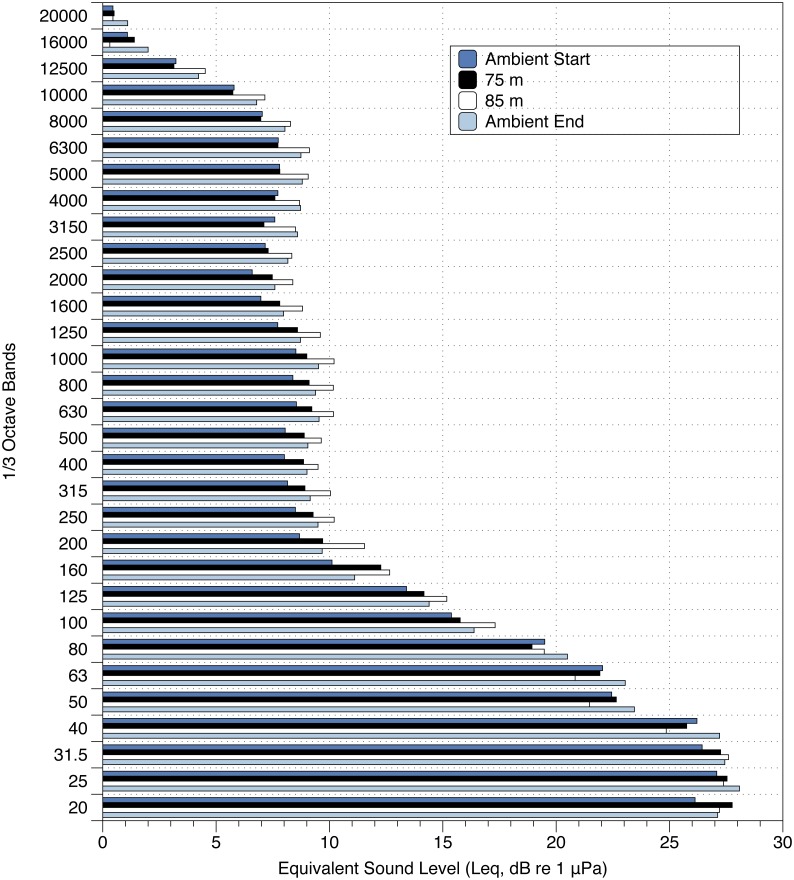
Equivalent Sound Level measurements at 1/3 octave bands for surveys with and without the eBee UAS. Equivalent Sound Level measurements at 1/3 Octave bands (dB re 20 µPa 1 m) for surveys with and without the eBee UAS. At 75 and 85 m, the eBee circled the UMIK-1.

Ambient sound measurements revealed significant variation in sound levels not associated with the presence of the UAS across 1/3 octave bands (Fig.  5). In all cases, equivalent sound levels were low (less than 30 dB re 1 uPa @ 1 m), and the presence of the UAS did not appear to contribute consistently to variation in soundscape levels across 1/3 octave bands, regardless of the survey altitude (Fig. 5). In some cases, equivalent levels were higher during control periods, and in other cases the opposite relationship was found (Fig. 5). The largest variation was a slight increase in ambient noise across all 1/3 octave bands, visible in ambient recordings done before and after the eBee trials. These data indicate that on a calm day, noises from the UAS are approaching ambient levels in an isolated field.

**Figure 6:**
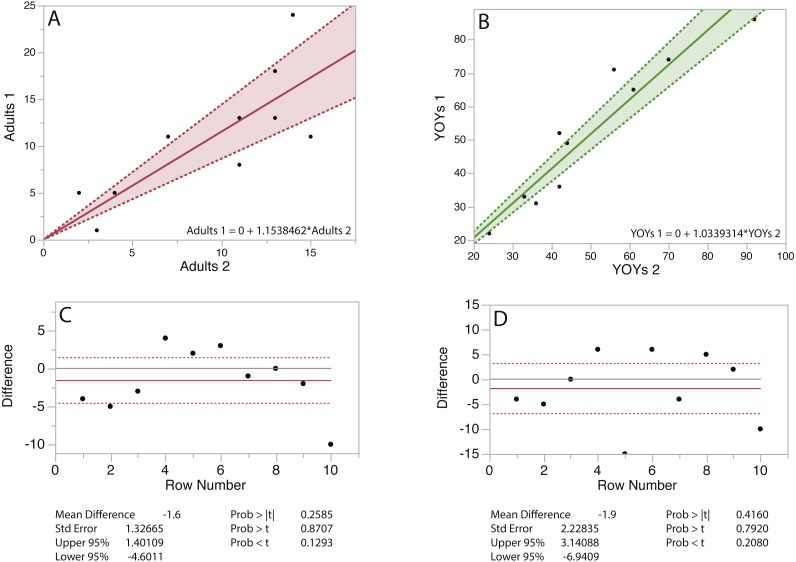
Linear regressions and matched pair *t*-tests for grey seals counted random quadrats on consecutive flights at Hay Island, Nova Scotia, Canada. Linear regressions (A & B) and matched pair *t*-tests (C & D) for adult and young-of-the-year (YOY) grey seals (B) counted in 10 random 50 m^2^ quadrats on 2 consecutive flights at Hay Island Nova Scotia, Canada.

**Figure 7:**
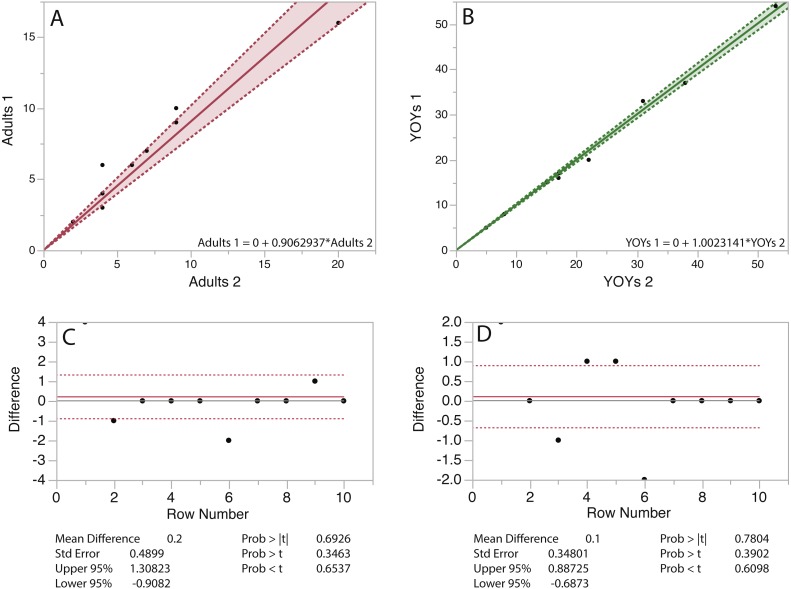
Linear regressions and matched pair *t*-tests for grey seals counted in random quadrats on consecutive flights at Saddle Island, Nova Scotia, Canada. Linear regressions (A & B) and matched pair *t*-tests (C & D) for adult and young-of-the-year (YOY) grey seals counted in 10 random 50 m^2^ quadrats on 2 consecutive flights at Saddle Island Nova Scotia, Canada.

The results of seal counts are presented in Figs. 6 and 7. At Hay Island adult and YOY counts in 10 random quadrats from consecutive flights did not vary significantly. The regressions for both adults and YOYs exhibits slopes approaching 1 with *R*^2^ values >0.80. The matched pair *t*-test was also not significant. The same is true for UAS flights at Saddle Island (Fig. 7). Regressing the number of adults and pups counted in random quadrats from consecutive flights also had slopes approaching 1 and high *R*^2^ values >0.80.

**Figure 8:**
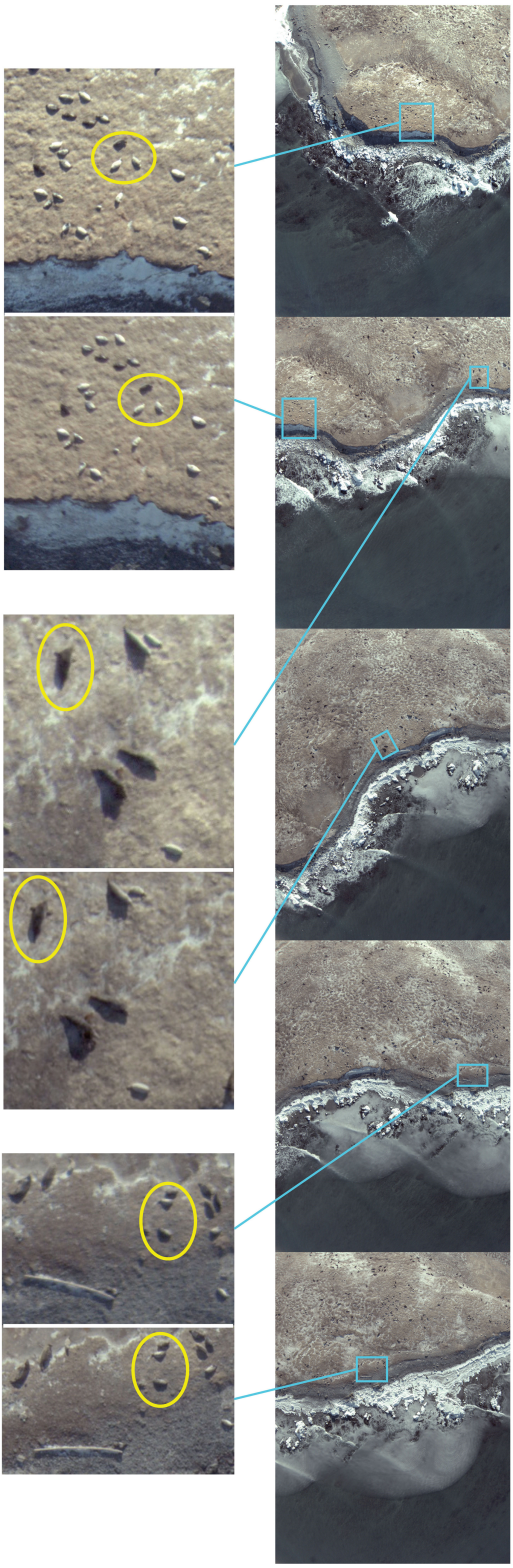
Assessments of consecutive images during initial UAV over-flight at Hay Island, Nova Scotia, Canada. Yellow ovals indicate example groups of seals imaged sequentially and assessed for movements and changes in posture.

**Figure 9:**
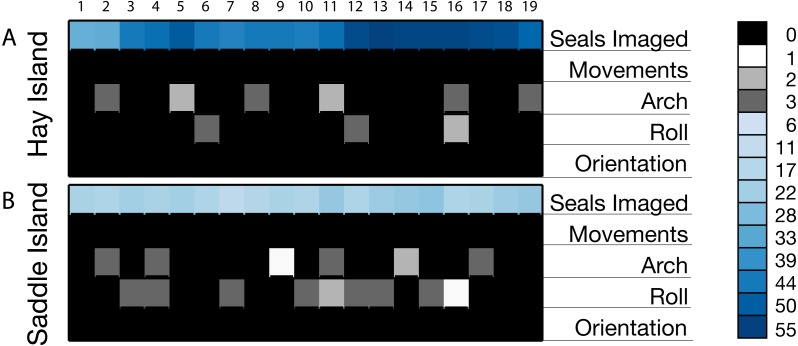
Cell plot of observed gray seals and behavioral responses in sequential images from overflights by senseFly eBee UAS at two gray seal colonies in NS, Canada. Cell plots of total seals assessed per image pair comparisons along with the total numbers of seals per image comparison that exhibited movements (>2 body lengths), body arches, body rolls or changes in orientation. Numbers along the top horizontal axis represent sequential image comparisons; the vertical color bar represents the total number of seals per comparison.

We assessed a series of 20 sequential images (19 comparisons between sequential images) along the initial flight tracks at each colony for changes in animal orientation or posture. For Hay Island, an average of 47 seals per image pair comparison were assessed for movements (>2 body lengths) body arching, rolling over, or changes in their orientation, resulting in a total of 889 seals examined. In these assessments, only 12 of 889 seals arched or rolled during imaging. For Saddle Island, an average of 21 seals per image pair comparison were assessed for movements (>2 body lengths) body arching, rolling over, or changes in their orientation, resulting in a total of 400 seals examined. In these assessments, only 21 of 400 seals arched or rolled during imaging. Figure 8 illustrates a cell plot matrix of the total number of seals assessed per image pair, as well as the number of seals that moved (>2 body lengths) arched, rolled, or changed orientation in each of these comparisons for both study locations. In these plots, numbers 1 through 19 represent sequential image pair comparisons. At both locations, no seals exhibited movements or changes in orientation, and no obvious patterns in arching or rolling are evident. Figure 9 illustrates a subset of consecutive images for the first line of the first flight at Hay Island. In these figures, highlighted example groups of seals were imaged repeatedly during a fly over with no evidence of movement, changes in orientation or posture of animals evident.

## Discussion

The use of UAS for wildlife monitoring is growing, and these platforms present significant opportunities to reduce costs (Mailey, 2013) and human risk (Sasse, 2003). Furthermore, the results of the present study indicate that small fixed-wing UAS can also provide improvements over traditional methods in terms of reduced disturbance of animals. These results directly address recent recommendations for research into disturbance of marine mammals through the use of UAS (Smith et al., 2016).

The results of UAS counts and image assessments indicate that both adult gray seals and pups do not react overtly to over-flights with this small fixed-wing platform. The number of gray seal adults and YOY at both survey locations were statistically similar between consecutive flights, indicating that animals were not flushed from habitats during surveys. The largest deviations were found with adults, likely indicating natural movements of these more mobile animals within the colony.

**Figure 10:**
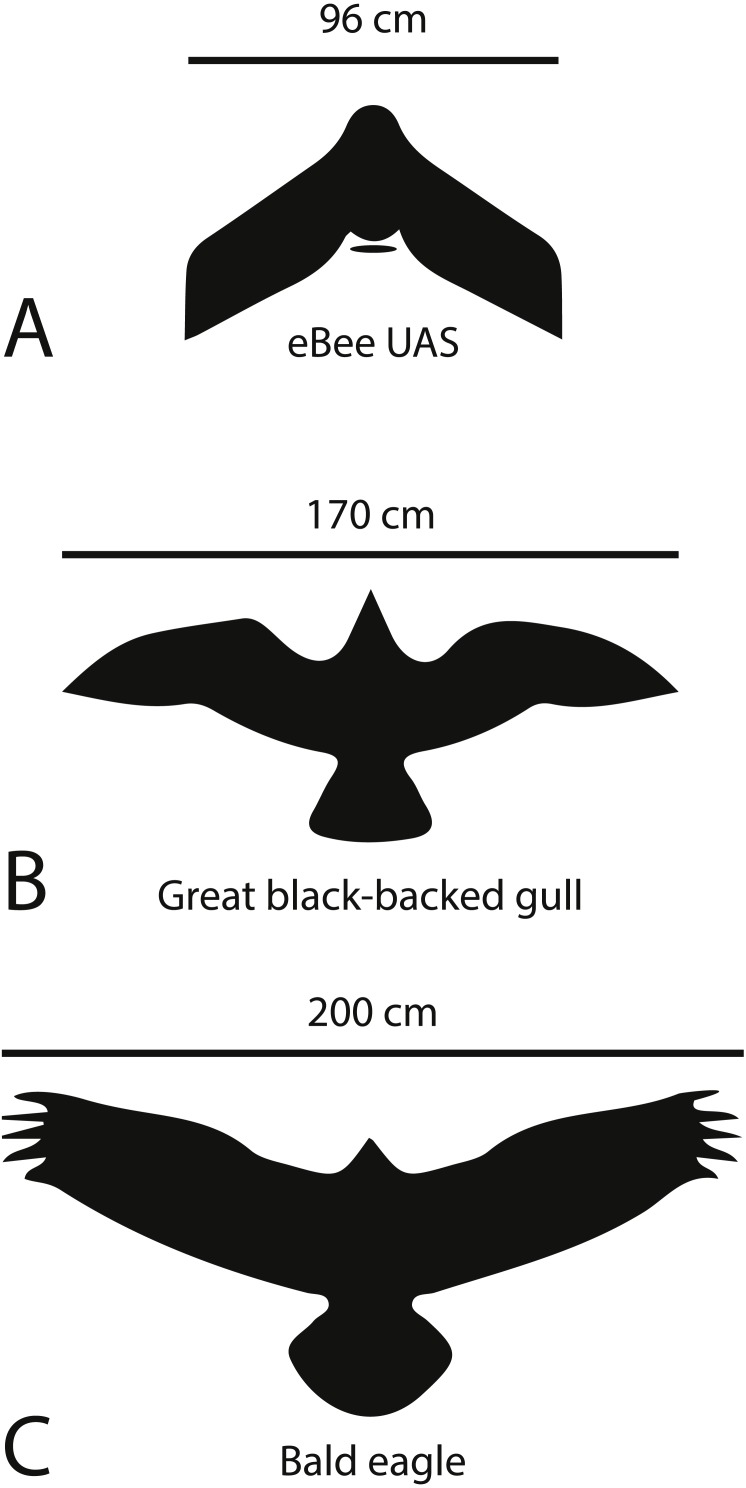
Silhouettes and wing spans of predatory birds in comparison to the eBee UAS. Scaled silhouettes and typical wing spans of predatory birds in the study region in comparison to the eBee UAS.

Detailed examinations of images taken sequentially revealed that seals did not react to and may not have noticed the UAS during flights. This is not surprising, considering the eBee’s sound production is essentially equivalent to ambient levels in low-noise environments, and that the airframe has a smaller silhouette (Fig. 10) than predatory birds frequently encountered in the study areas. Gray seals at breeding colonies are likely habituated to fly-overs by numerous bald eagles and great black-backed gulls soaring at similar altitudes to our UAS surveys. Both of these bird species are also seen with broken wings and beaks at seal breeding colonies, indicative of close encounters with females guarding pups. Our analysis of images and seal counts was also consistent with concurrent ground observations, where seals did not react overtly to fly-overs of the UAS. Similar studies with large mammals and small fixed-wing UAS also revealed no indication of disturbance (Vermeulen et al., 2013).

Traditional methods for assessing pinnipeds at haulouts often rely on humans walking around or through groups of animals or through the use of larger occupied aircraft for flyovers. Military occupied aircraft are known to cause considerable disturbance of wildlife, especially young or naïve animals (Lawler et al., 2005) and coastal animals such as pinnipeds can be startled and stampeded in the water by quieter aircraft used in wildlife surveys (helicopters and fixed-wing aircraft) if flown too low (Richardson et al., 1995).

It should be noted that ambient noise at shoreline locations encompasses a large range of sound sources including waves breaking and the sound of animals such as seabirds and pinnipeds (Deane, 2000). Considering this, ambient noise conditions at both Hay and Saddle Island would be much higher than in our test location, which greatly decreases the likelihood that gray seals could detect acoustically the UAS in flight while at a colony. Gray seals have good in-air hearing, with thresholds as low as 20 dB re 1 uPa peak equivalent sound levels at frequencies between 4 and 11 kHz (Ruser et al., 2014). Below 4 kHz, their hearing degrades rapidly by almost 40 dB per octave. Above 11 kHz, gray seal hearing thresholds ranged between 30 and 40 dB re 1 uPa (Ruser et al., 2014). In our acoustic measurements, the eBee (and ambient noise levels) only exceeded Leq values of 20 dB at frequencies below 40 Hz (Fig. 6). However, measurements of comparable peak equivalent sound levels for the eBee circling at 75 m ranged as high as 50 dB re 1 uPa across 1/3 octave bands. These peak measurements indicate the eBee might be detectable by gray seals at frequencies above 4 kHz in extremely quiet conditions, where the sounds of the UAS exceed ambient noise levels by some 12–26 dB re 1 uPa (the known critical ratios of phocid seals across our 1/3 octave bands, see Southall, Schusterman & Kastak, 2003). Considering the above, the present study reveals that small fixed-wing UAS are a good alternative to occupied aircraft in terms of reduced acoustic disturbance.

Comparing the costs of UAS-based wildlife surveys with occupied aircraft approaches is challenging due to differences in aircraft endurance, configuration and the status of operator (e.g., government vs contractor). As such, few if any direct comparisons exist. One example, focused on comparing UAS-based population surveys of sandhill cranes vs occupied aircraft, revealed that UAS costs ($2,600) were about 60% of the costs for government owned aircraft ($4,300), and more than an order of magnitude cheaper than the projected $35,000 private contractor costs (Mailey, 2013). This means that small UAS are accessible to regional agencies and local governmental bodies, including First Nations or indigenous governments, that may be interested in conducting population assessments of their own without incurring large infrastructure costs.

In the present study, operating costs for UAS were also lower than the traditional approach. Fixed contract prices for helicopter surveys in the study region can cost up to $2,200 USD per hour, and costs for fuel and pilot expenses increase this by approximately $350 USD per day. Typical small UAS flights are short, and costs are usually allocated on a per flight basis. Including platform overhead (approx. $500 USD per flight) and pilot/observer costs ($200 USD per day), data acquisition via small UAS is much more affordable. In fact, the traditional costs to survey both locations would allow for the purchase of two new eBee systems at current pricing (October, 2016) and provide imagery comparable to traditional means (Hammill et al., 2017; Johnston et al., 2017).

### Caveats and considerations

The present study indicates that no overt behavioral reactions occur for gray seals and YOY seals during small fixed-wing UAS flyovers. While this provides strong evidence of limited or negligible disturbance, it does not capture potential cryptic physiological effects of flights if the aircraft are indeed detected by seals. For example, a recent study found that black bears (*Ursus americanus*) exposed to small multirotor UAS did not react behaviorally, but responded physiologically through spikes in heart rate (Ditmer et al., 2015). Furthermore, social context and previous experience of animals may result in sensitization to UAS, as shown with some seal colonies in the UK (Pomeroy, O’Connor & Davies, 2015). At present it remains unclear how much human interactions occurs at the colonies assessed in the present study, and future research is required to better assess how human habituation may influence reactions to drone overflights.

Our UAS was acoustically unobtrusive and presented a diminutive aerial silhouette during flyovers in comparison to the commonly encountered predatory birds such as eagles and gulls found in the region. However, at lower altitudes the UAS could cause disturbance of animals, as both eagles and gulls are known to attack and feed on abandoned or starveling pups at gray seal colonies. These types of encounters have been reported at other pinniped breeding colonies. For example, kelp gulls are known to attack and blind Cape fur seal pups in Namibia (Gallagher, Staaterman & Dreyer, 2015). The UAS shape may also factor into the response of pinnipeds to low overflights. For example, UAS with delta-wing airframes (such as the eBee used in the present study) elicit stronger flight responses in seabirds than canard or glider-type airframes, perhaps because they more closely resemble the shape of predatory birds (McEvoy, Hall & McDonald, 2016).

Finally, there is great potential for species-specific responses to both acoustics and visual disturbance, and care should be taken when applying our results to other species. Further research is required to fully assess cryptic or contextual reactions to UAS surveys to fully establish their long-term effects on wildlife.

## Conclusions

The results of the present study reveal that the level of disturbance from small fixed-wing UAS surveys of gray seals breeding colonies is negligible, especially when compared to occupied aircraft surveys conducted at pinniped haulouts. Indeed, our results provide no indication that the animals detected the presence of the aircraft during surveys, possibly due limited audibility, small size and habituation to over-flight stimuli from large predatory birds in the area. Considering these results, and the potential reductions in cost and risks associated with traditional aerial survey approaches, we suggest that fixed-wing UAS-based approaches should be considered amongst best practices for assessing gray seal colonies.

##  Supplemental Information

10.7717/peerj.4467/supp-1 Supplemental Information 1 Acoustic measurements Equivalent sound level measurements for Arona et al.

## Additional Information and Declarations

### Field Study Permissions

The following information was supplied relating to field study approvals (i.e., approving body and any reference numbers): All observational flights were conducted according to Canadian small UAS rules (Exemption from Sections 602.41 and 603.66 of the Canadian Aviation Regulations, see http://www.tc.gc.ca/civilaviation/regserv/affairs/exemptions/docs/en/2880.htm) and under authorization by Fisheries and Oceans, Canada. All UAS flights for acoustic measurements were made under an FAA section 333 Exemption 12656 and associated blanket Certificate of Authorization (COA) awarded to Duke University.

### Data Availability

The following information was supplied regarding data availability: Hay Island orthomosaic: http://www.gigapan.com/gigapans/184322. Saddle Island Orthomosaic: http://www.gigapan.com/gigapans/184294.

## References

[ref-1] Anderson K, Gaston KJ (2013). Lightweight unmanned aerial vehicles will revolutionize spatial ecology. Frontiers in Ecology and the Environment.

[ref-2] Bevan E, Wibbels T, Najera B (2015). Unmanned aerial vehicles (UAVs) for monitoring sea turtles in near-shore waters. Marine Turtle Newsletter.

[ref-3] Deane GB (2000). Long time-base observations of surf noise. Journal of the Acoustical Society of America.

[ref-4] Ditmer MA, Vincent JB, Werden LK, Tanner JC, Laske TG, Iaizzo PA, Garshelis DL, Fieberg JR (2015). Bears show a physiological but limited behavioral response to unmanned aerial vehicles. Current Biology.

[ref-5] Durban JW, Fearnbach H, Barrett-Lennard LG, Perryman WL, Leroi DJ (2015). Photogrammetry of killer whales using a small hexacopter launched at sea. Journal of Unmanned Vehicle Systems.

[ref-6] Elarab M, Ticlavilca AM, Torres-Rua AF, Maslova I, McKee M (2015). Estimating chlorophyll with thermal and broadband multispectral high resolution imagery from an unmanned aerial system using relevance vector machines for precision agriculture. International Journal of Applied Earth Observation and Geoinformation.

[ref-7] Fretwell PT, Trathan PN (2009). Penguins from space: faecal stains reveal the location of emperor penguin colonies. Global Ecology and Biogeography.

[ref-8] Gallagher AJ, Staaterman ER, Dreyer N (2015). Kelp gulls prey on the eyes of juvenile Cape fur seals in Namibia. African Journal of Marine Science.

[ref-9] Hammill MO, Dale J, Stenson GB, Den Heyer C, Gosselin J-F, Johnston DW (2017). Comparison of methods to estimate grey seal pup production at different colonies. DFO Canadian Science Advisory Secretariat. Research Document.

[ref-10] Hodgson A, Kelly N, Peel D (2013). Unmanned Aerial Vehicles (UAVs) for surveying marine fauna: a dugong case study. PLOS ONE.

[ref-11] Inoue J, Curry JA (2004). Application of aerosondes to high-resolution observations of sea surface temperature over Barrow Canyon. Geophysical Research Letters.

[ref-12] Johnston DW, Dale J, Murray KT, Josephson E, Newton N, Wood S (2017). Comparing occupied and unoccupied aircraft surveys of wildlife populations: assessing the gray seal (*Halichoerus grypus*) breeding colony on Muskeget Island, USA. Journal of Unmanned Vehicle Systems.

[ref-13] Kardous CA, Shaw PB (2014). Evaluation of smartphone sound measurement applications. Journal of the Acoustical Society of America.

[ref-14] Koh LP, Wich SA (2012). Dawn of drone ecology: low-cost autonomous aerial vehicles for conservation. Tropical Conservation Science.

[ref-15] Koski WR, Allen T, Ireland D, Buck G (2009). Evaluation of an unmanned airborne system for monitoring marine mammals. Aquatic Mammals.

[ref-16] Krebs CJ, Hickman GC, Hickman SM (1994). Ecology: the experimental analysis of distribution and abundance.

[ref-17] Lancia RA, Kendall WL, Pollock KH, Nichols JD, Braun CE (2005). Estimating the number of animals in wildlife populations. Techniques for wildlife investigations and management.

[ref-18] Lawler JP, Magoun AJ, Seaton CT, Gardner CL, Boerjte RD, Ver Hoef JM, Del Vecchio PA (2005). Short-term impacts of military overflights on caribou during calving season. Journal of Wildlife Management.

[ref-19] Linchant J, Lisein J, Semeki J, Lejeune P, Vermeulen C (2015). Are unmanned aircraft systems (UASs) the future of wildlife monitoring? A review of accomplishments and challenges. Mammal Review.

[ref-20] Mailey C (2013). Are UAS more cost effective than manned flights? Arlington: AUVSI. http://www.auvsi.org/are-uas-more-cost-effective-manned-flights.

[ref-21] Mancini F, Dubbini M, Gattelli M, Stecchi F, Fabbri S, Gabbianelli G (2013). Using Unmanned Aerial Vehicles (UAV) for high-resolution reconstruction of topography: the structure from motion approach on coastal environments. Remote Sensing.

[ref-22] McEvoy JF, Hall GP, McDonald PG (2016). Evaluation of unmanned aerial vehicle shape, flight path and camera type for waterfowl surveys: disturbance effects and species recognition. PeerJ.

[ref-23] McMahon CR, Howe H, Van den Hoff J, Alderman R, Brolsma H, Hindell MA (2014). Satellites, the all-seeing eyes in the sky: counting elephant seals from space. PLOS ONE.

[ref-24] Moxley JH, Bogomolni A, Hammill MO, Moore KMT, Polito MJ, Sette L, Sharp WB, Waring GT, Gilbert JR, Halpin PN, Johnston DW (2017). Google haul out: earth observation imagery and digital aerial surveys in coastal wildlife management and abundance estimation. Bioscience.

[ref-25] Pater LL, Grubb TG, Delany DK (2009). Recommendations for improved assessment of noise impacts on wildlife. The Journal of Wildlife Management.

[ref-26] Pomeroy P, O’Connor L, Davies P (2015). Assessing use of and reaction to unmanned aerial systems in gray and harbor seals during breeding and molt in the UK 1. Journal of Unmanned Vehicle Systems.

[ref-27] Richardson WJ, Greene CR, Malme CI, Thomson DH (1995). Marine mammals and noise.

[ref-28] Ruser A, Dähne M, Sundermeyer J, Lucke K, Houser DS, Finneran JJ, Driver J, Pawliczka I, Rosenberger T, Siebert U (2014). In-air evoked potential audiometry of grey seals (*Halichoerus grypus*) from the North and Baltic Seas. PLOS ONE.

[ref-29] Sasse DB (2003). Job-related mortality of wildlife workers in the United States, 1937–2000. Wildlife Society Bulletin.

[ref-30] Seymour AC, Dale J, Hammill M, Halpin PN, Johnston DW (2017a). Automated detection and enumeration of marine wildlife using unmanned aircraft systems (UAS) and thermal imagery. Scientific Reports.

[ref-31] Seymour AC, Ridge JT, Rodriguez AB, Newton E, Dale J, Johnston DW (2017b). Deploying fixed wing Unoccupied Aerial Systems (UAS) for coastal morphology assessment and management. Journal of Coastal Research.

[ref-32] Smith CE, Sykora-Bodie ST, Bloodworth B, Pack SM, Spradlin TR, LeBoeuf NR (2016). Assessment of known impacts of unmanned aerial systems (UAS) on marine mammals: data gaps and recommendations for researchers in the United States. Journal of Unmanned Vehicle Systems.

[ref-33] Southall BL, Schusterman RJ, Kastak D (2003). Auditory masking in three pinnipeds: aerial critical ratios and direct critical bandwidth measurements. Journal of the Acoustical Society of America.

[ref-34] Sykora-Bodie ST, Bezy V, Johnston DW, Newton E, Lohmann KJ (2017). Quantifying nearshore sea turtle densities: applications of unmanned aerial systems for population assessments. Scientific Reports.

[ref-35] Vermeulen C, Lejeune P, Lisein J, Sawadogo P, Bouché P (2013). Unmanned aerial survey of elephants. PLOS ONE.

[ref-36] Zhang C, Kovacs JM (2012). The application of small unmanned aerial systems for precision agriculture: a review. Precision Agriculture.

